# Texas Sour Orange Juice Used in Scaffolds for Tissue Engineering

**DOI:** 10.3390/membranes8030038

**Published:** 2018-07-04

**Authors:** Mandana Akia, Nataly Salinas, Cristobal Rodriguez, Robert Gilkerson, Luis Materon, Karen Lozano

**Affiliations:** 1 Department of Mechanical Engineering, University of Texas Rio Grande Valley, Edinburg, TX 78539, USA; mandana.akia@utrgv.edu (M.A.); nataly.salinas01@utrgv.edu (N.S.); 2 Department of Biology, University of Texas Rio Grande Valley, Edinburg, TX 78539, USA; cristorod13@gmail.com (C.R.); robert.gilkerson@utrgv.edu (R.G.); luis.materon@utrgv.edu (L.M.)

**Keywords:** centrifugal spinning, polyhydroxybutyrate, membrane, biomedical

## Abstract

Fine fibers of polyhydroxybutyrate (PHB), a biopolymer, were developed via a centrifugal spinning technique. The developed fibers have an average diameter of 1.8 µm. Texas sour orange juice (SOJ) was applied as a natural antibacterial agent and infiltrated within the fibrous membranes. The antibacterial activity against common Gram-positive and Gram-negative bacteria (*Staphylococcus aureus* and *Escherichia coli*, respectively) was evaluated as well as cell adhesion and viability. The PHB/SOJ scaffolds showed antibacterial activity of up to 152% and 71% against *S. aureus* and *E. coli*, respectively. The cell studies revealed a suitable environment for cell growth and cell attachment. The outcome of this study opens up new opportunities for fabrication of fibrous materials for biomedical applications having multifunctional properties while using natural agents.

## 1. Introduction

Wound management requires the use of wound dressings that could create suitable conditions to encourage the healing process, while providing external protection [1]. In recent years, the study of nanofiber-based membranes for wound care applications has been intensified. The high surface area and nanoporosity of these membranes provide a promising material to be used as an antibacterial agent and as a scaffold for tissue regeneration [2,3]. Most of the reported studies have developed the nanofiber membranes using the electrospinning method, patented in 1902 by Cooley [3,4,5,6,7,8,9]. Recently, the Forcespinning^®^ method patented in 2014 by Lozano and Sarkar [10], developed to scale up the fabrication of fine fiber membranes, has gained increasing attention in the biomedical field [11,12,13,14]. The preferred polymeric systems for wound care management have been PVP (polyvinyl pyrrolidone), PEO (polyethylene oxide), PLA (polylactic acid), PEG (polyethylene glycol), PVA (polyvinyl alcohol), PLGA (polylactide-co-glycolic acid), and PCL (polycaprolactone), to mention some [3,15].

Results from studies conducted in the development of wound dressing materials with antibacterial activity have mostly focused on the functionalization of polymeric systems with antibacterial agents (such as Beta-lactams, Aminoglycosides, Quinolones, Sulphonamides, Tetracyclines, and Glycopeptides) [16,17,18,19] and utilization of metal nanoparticles (such as Ag [20,21,22,23,24,25,26], ZnO [27,28], CuO [29,30], and others like TiO_2_ [31,32,33], quantum dots [34,35], etc.). However, concerns about resistance to antibiotics and the toxicity of metal nanoparticles [36] have resulted in the need to design a road map for exploring new antimicrobial agents. Natural antipathogenic agents are gaining interest in the development of nanofiber-based membranes to reduce the risk of infections [37]. Zhang et al. have examined the antibacterial activity of different electrospun fiber mats containing various plant extracts such as Green tea, Chamomile, and Cinnamon. The effect of some common bacteria—*S. aureus* and *E. coli*—was evaluated on the fabricated fibrous materials [38]. Yao et al. incorporated *Centella asiatica* (CA), a kind of herbal medicine, into electrospun gelatin membranes (EGC). In vitro studies in a rat model demonstrated higher collagen deposition within the EGC membranes when compared with other applied treatments [39]. Chan et al. added a Chinese herbal extract (baicalein, BAI) into silk fibroin protein (SFP) mixed with PVP, obtained an SFP/PVP/BAI nonwoven mat, and evaluated its performance as wound healing materials. The fabricated electrospun mats promoted cell regeneration in the wound coupled with enhanced antibacterial activity against *S. aureus* [40]. Other studies have used naturally derived agents such as soy protein, silk fibroin, zein [41,42], thymol, aloe vera, emu oil, and honey [15,43] to develop polymeric-based fiber membranes. Among the different natural antibacterial compounds incorporated into electrospun fibers, honey has gained special attention in wound healing applications. The antibacterial ability of honey has been attributed to its low pH (3.5–4), which can modify the alkaline environment of chronic wounds. Although it has been shown that reducing the pH within the wound area improves the healing process, only few studies have followed this strategy to control bacterial growth [3].

Sour orange or bitter orange (pH of 2 to 2.6) is quite popular in Asian countries where all parts of this fruit have traditionally been utilized as herbal medicine [44,45]. In general, citrus fruits including sour orange are mainly composed of organic acids and sugars, though also have bioactive compounds, such as hydrocinnamic acid, ferulic acid, hesperidin, cyanidin glucoside, vitamin C, carotenoid, and naringin (responsible for the bitter taste), which provide special health benefits [46]. Antibacterial properties of different kinds of citrus species have been investigated mostly focusing on the antibacterial activity of peel essential oils [45,47,48,49]. It was demonstrated that different sweet and sour orange cultivars may show different antibacterial behavior [45].

Polyhydroxybutyrate (PHB) is a synthetic biodegradable and biocompatible polymer which has shown promise in the biomedical field, specifically in tissue-engineering-related studies [37,50,51,52]. In this study, PHB fine fiber membranes were developed via the Forcespinning^®^ process. The developed nonwoven fiber membranes were coated with sour orange juice. Antibacterial activity and cell growth studies were conducted.

## 2. Materials and Characterization Techniques

### 2.1. Materials and Methods

Polyhydroxybutyrate biopolymer (PHB) with a granule size of 5 mm and molecular weight of 550,000 was purchased from Goodfellow (Cambridge Ltd., Cambridge, UK). The required needles and syringes for the fiber spinning process were obtained from Fisher Scientific. Sour orange juice (SOJ) was squeezed from South Texas sour oranges. The juice was collected in vials and left aside for at least three days; the suspended particles settled and the clear phase was used.

### 2.2. Fabrication of Fine Fiber Membranes

Polymer solutions containing PHB (11 wt %) in chloroform were prepared by placing the mixture in an oil bath at 60 °C for at least 5 h to get a homogenous solution. The obtained solution was fed (1 mL) into a two-nozzle spinneret (30 G needle tips). The distance from needle tips to collectors was 14 cm. The fibers were spun at 5000–6000 rpm under a relative humidity (RH) of 45 to 50%. The spinning time was set for 2 min after which the fibers were collected in a nonwoven fashion using a metallic square frame. The obtained mats were vacuum-dried at 65 °C for at least 12 h to remove solvent remnants. The samples were then immersed in SOJ for 5 min and left to dry at room temperature for 24 h. The samples were then stored in plastic bags.

Scanning electron microscopy was conducted using a Sigma VP SEM, Carl Zeiss, Germany. Fiber morphology and diameter distribution was evaluated. The samples were gold sputtered utilizing a Denton’s Desk V deposition unit (Denton Vacuum, Moorestown, NJ, USA). Images were taken at a voltage of 1 kV. The average fiber diameter and histograms were analyzed using ImageJ (US National Institutes of Health, Bethesda, MD, USA) and Minitab^®^17 (Minitab Inc., State College, PA, USA) statistical software. For the statistical analysis, a *t*-test method with 95% confidence was employed.

### 2.3. Antibacterial Test and Dilution Method

The antibacterial test was conducted using the disk diffusion method and the dilution method. *E. coli* (ATCC 8739) and *S. aureus* (ATCC 6538) were seeded on Mueller-Hilton agar plates (Oxoid Ltd, Basingstoke, UK). The plates were incubated at a temperature of 37 °C for 24 h to promote the growth of the bacteria. For the disk diffusion method, the developed fiber membranes were cut using a Biopunch (Acuderm Inc., Fort Lauderdale, FL, USA) with a diameter of 5.5 mm. Control (pure PHB fiber membranes) and sour-orange-juice-coated PHB fiber membranes were evaluated. To perform the inhibition zone experiment, 25 mL of sterilized agar was added to each sterilized plate, followed by injecting a loop of the required bacterium. The fibrous mats were then placed in the plates. Six replicates were analyzed for each sample. Before analyzing the results, plates were incubated at a temperature of 37 °C for 24 h. For the dilution method, the fiber mats were cut as 1 square centimeter samples. A quantity of 10 µL of a 24-day bacterial suspension was inoculated into 10 mL of nutrient broth. Then, 100 µL was taken from the 10 mL broth containing the membranes and introduced into blanks of 9.9 mL to start a 10-fold dilution series. Aliquots of 100 µL from three different dilution levels were taken to inoculate triplicate plates of Mueller-Hilton agar. The inoculum was spread over the surface of the plates using L-shaped glass rods to further observe colony formation. The plates were then incubated at 37 °C from 24 h to 96 h and the surviving bacteria (average from three plates) were counted applying the spread plate procedure [53,54].

### 2.4. Cell Proliferation and Cell Viability (MTT Assay)

Immortalized NIH 3T3 mouse embryonic fibroblast cells were cultured in Dulbecco’s modified Eagle’s medium (DMEM) with 10% fetal bovine serum (FBS) and penicillin–streptomycin (100 units/mL) and maintained at a temperature of 37 °C for 48 h in an air atmosphere mixed with 5% CO_2_. Fiber mats of 1.0 cm × 1.0 cm were sterilized under ultraviolet light for at least 20 min and then placed in 6-well cell culture plates.

For the cell adhesion/viability experiments, 250,000 embryonic fibroblast cells (NIH 3T3) were deposited on the control and SOJ-coated PHB membranes. The samples were incubated at 37 °C under air mixed with 5% CO_2_ for 7 days. After incubating for 24 h, cells were stained with MitoTracker Red (MTR) to analyze the mitochondrial morphology, and stained with 4’, 6-diamino-2-phenylindole (DAPI) to analyze the cell nuclei. Samples were rinsed twice with phosphate-buffered saline (PBS) solution to remove nonadherent cells or stain residue, then fixed with 4% paraformaldehyde in PBS, and finally mounted with 50% glycerol in PBS. Cells were viewed via confocal laser scanning fluorescence microscopy using an Olympus FV10i microscope (Olympus America Inc., Center Valley, PA, USA). In order to observe cell density, a quantitative analysis was conducted from 6 images taken randomly from the sample area (*n* = 3 independent experiments). All the values were expressed based on statistical analysis via Student’s t-test measuring the mean ± standard error from the quantitative number of the cell count per image in a 212 × 212 (µm^2^) area.

In the case of the cell viability studies, the PHB fibers (control) and SOJ-coated PHB fibers were examined using an MTT assay. The samples were placed in 6-well culture plates. A total of 250,000 embryonic fibroblast 3T3 cells were seeded onto the samples over the course of 7 days. Prior to the MTT assay analysis, coverslips and 6-well culture plates were replaced with new ones. For the MTT assay, 2 mL of a 0.5 mg/mL solution of MTT (3-(4,5-Dimethylthiazol-2-yl)-2,5-Diphenyltetrazolium Bromide) in cell culture media was added to each well, and allowed to incubate for 2 h. After the incubation step, media were removed and samples were rinsed with PBS to remove nonadherent cells or MTT residue. Then, 1200 μL of dimethyl sulfoxide (DMSO) was added to each sample followed by slow agitation for 30 min to separate the cells from the mat. The resulting solutions were transferred into a 96-well plate and analyzed on a Bio Rad iMark™ microplate reader at a 595 nm wavelength.

## 3. Results and Discussion

### 3.1. Fiber Morphology

**Figure 1:**
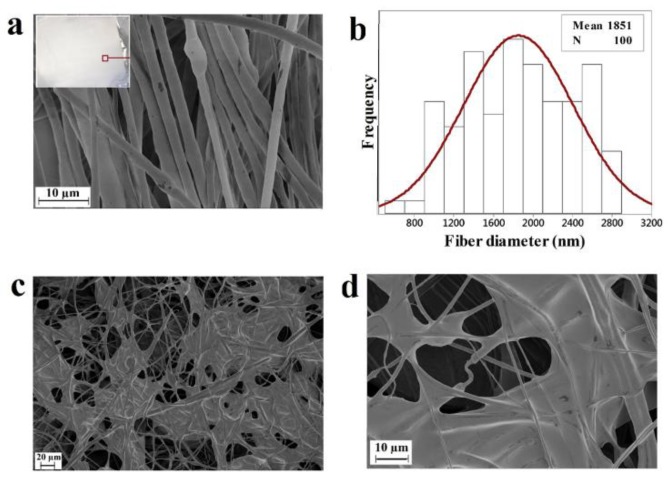
Scanning electron micrographs of polyhydroxybutyrate (PHB) fibers (**a**) along with the fabricated fiber mat (upper inset), PHB fiber diameter distribution (**b**), and sour orange juice (SOJ)-coated PHB fibers membrane at different magnifications of 20 µm and 10 µm (**c**,**d**).

Figure 1 shows scanning electron micrographs of the developed fiber membranes and the statistical analysis of the fiber diameter. Figure 1a shows the developed PHB mat (upper inset) along with the SEM image of the PHB fibers. The developed fibers had rough surfaces resulting from the rapid evaporation of the solvent. The average fiber diameter was found to be 1.8 µm and its distribution is presented in Figure 1b. The surface morphology of the SOJ-coated PHB fibers is presented in Figure 1c,d which show a veil formation connecting the fibers. This veil is due to the absorbed SOJ. This result may indicate the ability of this structure to effectively trap the liquid agent and thereby provide a moist medium, which is considered as an encouraging parameter to enhance the healing process [1,55]. Some studies have reported that the formation of spider webbing and netting structures within fibrous mats (based on the SEM images) have promoted an increase in the number of reaction sites, resulting in the formation of hydrogen bonds and ultimately enhancing the physical properties and biological activity of the fibrous mats [55,56].

### 3.2. Antibacterial Results

**Figure 2:**
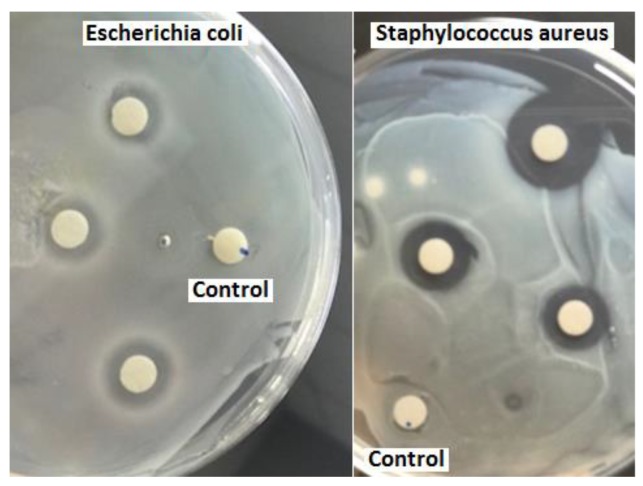
The inhibition zone activity of the PHB (control) and SOJ-coated PHB samples against *S. aureus* and *E. coli* bacteria; control samples are marked with blue dots. The diameter of the fibrous mats is 5.5 mm.

Figure 2 shows the results of the antibacterial analysis: three SOJ-coated PHB fiber mats 5.5 mm in diameter together with the PHB (control sample) mat are shown in the presence of *S. aureus* and *E. coli*. It can be clearly seen that the SOJ-coated PHB fiber mats have a clear ring around the mats (known as inhibition zone) depicting antibacterial activity while the control sample is uniformly covered with bacteria. The results show larger inhibition zones against the Gram-positive bacteria (*S. aureus*); however, the antibacterial activity against *E. coli* was also fair. These results are obtained in the absence of sterilization prior to evaluating the antibacterial effect—reported studies use UV [57] and autoclaving [26] sterilization prior to testing. The average inhibition zones (diameter of the clear ring including the 5.5 mm for the sample) for *S. aureus* and *E. coli* were 12 ± 2 and 8.2 ± 1.3 mm, respectively. The antibacterial properties of sour orange are attributed to its acidic nature [58]. The combination of SOJ with fine fiber structures with enhanced surface area further increases the opportunity to expose the antibacterial agent [3].

**Figure 3:**
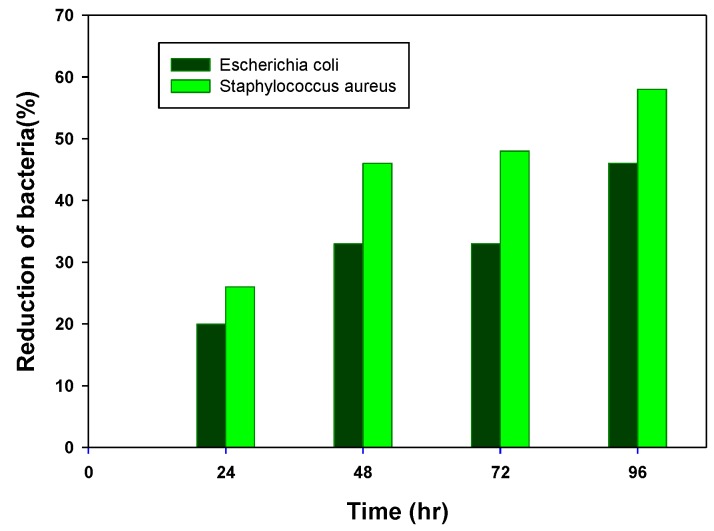
The rate of bacteria reduction in SOJ-coated PHB fibers membrane during 96 h of experiment, based on the dilution method.

Figure 3 shows the percentage of bacteria reduction based on the dilution method. It was calculated applying the formula of (A − B)/A×100 [57,59], where A and B are surviving bacteria for the control and SOJ-coated samples, respectively. The expressed results are based on averages from three plates.

### 3.3. Cell Proliferation and Viability

**Figure 4:**
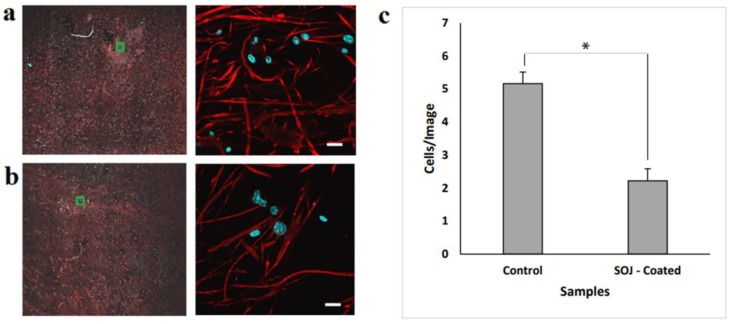
Cell adhesion and proliferation for PHB samples after 7 days. The figure shows map images (left) of PHB samples along with higher-magnification images (right) taken from the area within the green squares. (**a**) PHB mats as control samples; and (**b**) SOJ-coated PHB fiber mats. Cell nuclei were labeled with 4’, 6-diamino-2-phenylindole (DAPI) (blue), with MitoTracker Red labeling both mitochondria and PHB fibers (scale bar = 20 μm). (**c**) Comparison between the numbers of cells in the control and SOJ-coated PHB fiber membranes based on 6 images per sample (* *p*-value < 0.05).

Figure 4a,b show cellular distribution and proliferation on the fibrous membranes (cell nuclei labeled with DAPI (blue) and mitochondria and fibers labeled with MitoTracker (red)). Mitochondria are critically important for cellular metabolism [60]. The red fluorescent dye, MitoTracker Red CMXRos, accumulates in response to transmembrane potential across the mitochondrial inner membrane [34]. Cell proliferation over 7 days was quantified by analyzing DAPI-stained nuclei in both PHB fiber membrane samples (control and SOJ-coated).

The number of cells/image was generated from high-magnification images (6 images/sample) of the selected areas (Green Squares, Figure 4a,b) and is depicted in Figure 4c. A *p*-value of <0.05 was obtained from the confocal microscopy analysis, which means the results were significantly different, suggesting that SOJ does not increase cell viability on PHB. This may be attributable to the settling of the cells within the different layers of the mats. Various studies have reported that fibers with smaller diameters (e.g., nano-sized) favor cell adhesion when compared with larger-sized fibers (e.g., micro-sized). The average size of fibers in this study was 1.8 micrometers; however, the cells in both systems (the control and SOJ-coated samples) were still able to adhere and survive in these fiber mats. In other words, both samples displayed viable cells growing within the fiber matrix. Additionally, the high surface area of fiber mats coupled with high surface roughness could play a role in improving both the cell adhesion and proliferation [2,61].

**Figure 5:**
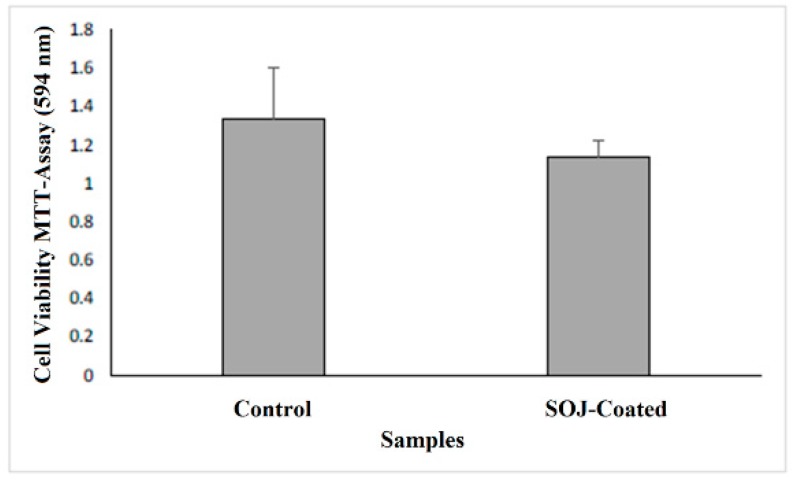
Comparison of cell viability (3T3 Mouse Embryonic Fibroblast) between PHB fibers (control) and SOJ-coated PHB fibers via MTT assay following 7 days of incubation, *n* = 3 experiments. (* *p*-value < 0.05)

Cell viability and biocompatibility were assayed by MTT as a complementary approach to the confocal imaging. Figure 5 shows MTT values for control PHB fiber membranes and SOJ–PHB fibers. In comparing control versus SOJ-coated PHB fibers, no significant difference was shown, indicating that SOJ does not negatively impact the ability of 3T3 fibroblasts to adhere and grow within PHB fiber matrixes. This is consistent with the presence of intact 3T3 cells shown on both samples via confocal microscopy (Figure 4a,b). The lower quantities of cells present on SOJ-coated fibers might be attributed to a small sample size. The confocal imaging and MTT assay results, taken together, suggest that SOJ coating allows cell adherence and viability. Upon addition of DMSO to the samples, the control mats were partially dissolved, while the SOJ-coated PHB mats remained unchanged. Comparison between the viability in control and SOJ-coated samples indicates that the cells are viable after 7 days.

## 4. Conclusions

To explore natural antibacterial agents for reducing infection in wound healing and treatment, SOJ was incorporated into PHB fibers. The wettability of the SOJ-coated PHB fiber structure (as depicted in SEM images) could allow it to serve as a suitable structure in tissue engineering applications. *Staphylococcus aureus* and *Escherichia coli* were used to evaluate the bactericidal activity of the fabricated membranes. As the antibacterial results revealed, the effect on *S. aureus* was stronger than that on *E. coli* (152% against 71%) for SOJ-coated PHB fibers. The dilution experiment demonstrated reduction rates of 58% and 48% for *S. aureus and E. coli*, respectively, during 5 days of running tests. Confocal imaging (Figure 4a,b) and MTT assay (Figure 5) together demonstrate that SOJ-incorporated fibers support cell adhesion and viability. To sum up, the antibacterial and cellular behavior (cell attachment and cell growth) of SOJ-coated PHB fibers combined with the biodegradability and biocompatibility of the PHB fiber membranes suggest potential applications of the fabricated membranes for tissue engineering. In the near future, the incorporation of SOJ on scaffolds with different fiber diameters can be investigated to further improve biomedical applications.
